# Can Network Linkage Effects Determine Return? Evidence from Chinese Stock Market

**DOI:** 10.1371/journal.pone.0156784

**Published:** 2016-06-03

**Authors:** Haishu Qiao, Yue Xia, Ying Li

**Affiliations:** College of Finance and Statistics, Hunan University, Changsha, China; East China University of Science and Technology, CHINA

## Abstract

This study used the dynamic conditional correlations (DCC) method to identify the linkage effects of Chinese stock market, and further detected the influence of network linkage effects on magnitude of security returns across different industries. Applying two physics-derived techniques, the minimum spanning tree and the hierarchical tree, we analyzed the stock interdependence within the network of the China Securities Index (CSI) industry index basket. We observed that that obvious linkage effects existed among stock networks. CII and CCE, CAG and ITH as well as COU, CHA and REI were confirmed as the core nodes in the three different networks respectively. We also investigated the stability of linkage effects by estimating the mean correlations and mean distances, as well as the normalized tree length of these indices. In addition, using the GMM model approach, we found inter-node influence within the stock network had a pronounced effect on stock returns. Our results generally suggested that there appeared to be greater clustering effect among the indexes belonging to related industrial sectors than those of diverse sectors, and network comovement was significantly affected by impactive financial events in the reality. Besides, stocks that were more central within the network of stock market usually had higher returns for compensation because they endured greater exposure to correlation risk.

## Introduction

In recent years, the application of complex network analysis to financial issues which kept increasing in popularity has attracted much interest from researchers both in physics and economics [1–4]. In particular, many scholars focus their studies on the stock networks for the rising significance of stock market comovements as a consequence of intensive economic globalization [5, 6]. The physics-derived data mining method, namely minimum spanning tree (MST) analysis which allows for unveiling clustering behavior within financial markets in a quite elucidative manner has been a standard technique to extract networks from correlation matrices in the realm of econophysics [7]. As a result, numerous investigations have been performed on recognition of the topological structure and statistical features of stock markets adopting the MST methodology on account of its robustness and simplicity [8, 9]. For example, researches exploring international stock market indices [10], EU stock market indices [11], as well as researches on individual stocks in national stock market of England, Germany, Turkey, Brazil and China [12–19].

Nevertheless, the aforementioned investigations generally obtain competing MST results due to the diversification of correlation coefficient matrices construction method. A vast body of empirical literature on stock market networks adopted the rolling correlation coefficient (RC) process to obtain correlation coefficient matrices [14–16], whereas it is currently universally acknowledged that the RC technique does not perform well for the case of financial high frequency data analysis. On one hand, using rolling window technique to construct stock networks may obtain multifarious results due to researchers’ specific option of parameters, namely the length and drift of the estimation window, thus undermining the objectivity and reasonability of the research conclusions to some extent [20]. On the other hand, given that the stock market comovements usually exhibit increased volatility virtually, the correlation coefficient estimate would be exposed to contortion caused by data heteroskedasticity and encounter severe upward bias, thereby resulting in misleading findings. In this study, we contribute to the extant literature by applying the cDCC MV-GARCH model suggested by Engle [21] to calculate dynamic conditional correlations, which not only considerably overcome the chaos in selecting parameters of the estimation window directly by offering full-sample correlation estimates, but are also anticipated to obtain higher robustness with respect to the heteroskedasticity matter[22,23].

Furthermore, massive research efforts of stock market network have been exerted to the topological structure and statistical feature of the network itself, while there have few studies associated with the intrinsic exploratory problems such as the effects of peculiar network topological properties on stock returns to date [24]. The absence of a verifiable relationship between stock market risk and accompanied returns generates problems for dominant asset pricing model analyses. Specifically, a strand of literature argues that interdependence among stock returns may partly originate from aggregate risk, which raises a noticeable question concerning whether future security returns could be interpreted by the dynamic market correlations [25, 26]. There is a need for further research by discussing the linkage effects between stock market network topological metrics to unveil how the underlying co-movement across local stocks affect their market performance, and to clarify whether stocks with larger centrality in the network usually acquire higher returns as they suffer from greater exposure to systematic risk.

It is apparent that research has traditionally concentrated on major developed economies while there have been few studies associated with the stock market network of emerging economies to date, and most attention is centered on the interdependence of the stock market across geographical borders but not through industry classifications. Hence, assessment of the comovement between industry indices in the context of Chinese stock market can fill the existing gap in the literature on financial network. Concerned about all the aforementioned reasons, we aim to use the CSI industry indices to obtain the classification taxonomy of Chinese stock market and then propose a network approach by combining dynamic conditional correlation, the MST method and the hierarchical tree analysis for elucidating the topological natures and connection structures of stock networks. Subsequently, we further investigate the stability of linkage effects among stocks’ network, by calculating the mean correlations and mean distances, as well as the normalized tree length of these indices. Afterwards, we estimate stocks’ network centrality to investigate whether the findings of industries are also appropriate for stocks, i.e. whether individual stocks in more central network locations are inclined to have higher returns.

The remainder of the paper is organized as follows. Section 2 and Section 3 discuss the empirical data and applied methodology. We then represent the main empirical results and analyses in Section 4, subsequently Section 5 summarizes and concludes the paper.

## Data Set

We unveil the underlying relationship of Chinese stock market interactions using three-tier China Securities Index (CSI) industry index data in S1 Text. The experimental data set utilized to build the networks includes daily closing prices offered by the WIND information database for indices at different levels which covers the following three time intervals respectively: January 04, 2002 to July 01, 2015 (one-tier industry), January 05, 2009 to July 01, 2015 (two-tier industry) and January 05, 2009 to June25, 2015 (three-tier industry). The indexes set examines the enormous market performance of China’s A-shares cosmos, including over 1200 stocks with complete data disclosure and traded on both Shenzhen and Shanghai exchanges, and is viewed as an authentic indicator able to outline the dynamic features of clusters in China’s stock market, and to provide helpful portfolio analysis instrument for investors. Moreover, the construction of CSI industry indices not only fully consult the worldwide common classification taxonomy standard, but also gives adequate consideration to distinctive characteristics of China’s economic circumstances. It includes progressively refined three-tier CSI Industry indices, and there are ten sub-indices in the one-tier industry index which reflect the following specific sectors: finance, industry, basic materials, energy, utilities, consumer goods, capital, information technology, telecommunications and health. In consequence, the CSI industry indices are sufficiently qualified as the representative of China’s stock market index.

In addition, an issue that needs to be addressed is that unlike an extensive literature which rectifies the option of rolling window parameters arbitrarily in terms of the significance of empirical results, the selection of samples in this study completely relies on the official data provided by the WIND information database, thus being more credible and realistic. The three-tier industry indices in our research and the respective symbols are presented in Table 1.

**Table 1:** Three-tier CSI Industry indices and respective symbols.

**One-tier Industry index**	**Code**	**One-tier Industry index name**	**Code**
**CSI 300 Financials Index**	CFI	CSI 300 Materials Index	CMI
**CSI 300 Telecommunication Services Index**	CTS	CSI 300 Information Technology Index	CIT
**CSI 300 Consumer Discretionary Index**	CCE	CSI 300 Utilities Index	CUI
**CSI 300 Health Care Index**	CHC	CSI 300 Energy Index	CEI
**CSI300Consumer Staples Index**	CCS	CSI 300 Industrials Index	CII
**Two-tier industry index**	**Code**	**Two-tier industry index name**	**Code**
**300 Retail**	RET	300 IT Software	ITF
**CSI 300 Banks Index**	CBI	300 Auto and Component	ACP
**300 Pharma & Biotech**	PBT	CSI 300 Materials	CMA
**300 IT Hardware**	ITH	CSI 300 Real Estate Index	CRE
**300 Food & Beverage**	FBA	300 Utilities	UTL
**300 Media**	MEA	300 Diversified financials	DFI
**300 Insurance**	INS	300 Capital Goods	CAG
**300 Energy**	ENG	CSI 300 Trans	CTR
**300 Durables& Apparel**	DAP		
**Three-tier Industry Index**	**Code**	**Three-tier Industry Index**	**Code**
**300 Multi Retail**	MRA	300 Household Durables	HOD
**300 Textiles & Apparel**	TEP	300 Trading Companies	TRC
**300 Beverages**	BEA	300 Food	FOD
**300 Specialty Retail**	SPR	300 Electric	ELR
**300 Pharm**	PHA	CSI 300 Real Estate Index	REI
**300 Chemicals**	CHA	300 Metals & Mining	MEM
**300 Commercial Banks**	COB	300 Capital Markets	CAM
**300 Marine**	MAN	300 Airlines	AIR
**300 Media**	MEA	300 Auto Components	AUC
**300 Insurance**	INS	300 Defense	DES
**300 Electrical Equip**	ELE	300 Trans-infrastructure	TRF
**300 Oil & Gas & Coal**	OGC	300 Machinery	MAI
**300 Construction Materials**	COM	300 Construction	COU
**300 Computers**	COT	300 Water Utilities	WAU

## Method

### Network construction

#### Minimum spanning tree

In this paper, we utilize Kruskal’s algorithm to construct a minimum spanning tree (MST) to examine the extent and evolution of interdependence among CSI industry indices. A brief description of MST construction is proposed by Mantegna [27]:

Step 1. Regard each index as node and linkage effect as edge in a network. Consider each node as an isolated branch, and sort the edges by their weights which denote the degree of linkage effects among indices.
Step 2. Pass through the network once and search an edge with the minimum weight and ensure no closed loop is created. This edge is added to the minimum spanning tree set if all the requirements are met. Otherwise, continue to traverse the network to seek a next edge with the minimum weight.
Step 3. Recursively repeats the former steps, until n-1 edges have been identified (if the network has n nodes, the minimum spanning tree should have n−1 edges since there are no closed loops in MST). Then, the searching process terminates and the network’s minimum spanning tree is obtained by selecting the most important correlations between the index returns.

#### Subdominant ultra-metric space method

Ultra-metric space method is used to describe the hierarchical structure of complex systems. Assuming there is a collection in which distances between any two elements can be defined, we can attain several ultra-metric spaces by dividing the collection. Let X be a collection, if all x, y, z in d: M × M → R meet the following conditions,

1. d(x, y)≥0
2. d(x, y) = 0,x = y
3. d(x, y) = d(y, x) (symmetry)
4. d(x, z)≤max{d(x,y),d(y,z)} (triangle inequality)

we call this metric space an ultra-metric space.

Subdominant ultra-metric space is a special form of the ultra-metric space, which was defined by Bayod et al. as follows: l (X, d) is a metric space and S is a collection whose elements are ultra-metric and all meet the condition: for each x, y ∈ X and p ∈ S, p (x, y) ≤ d (x, y). Taking supS as the supremum of elements in S, then (X, supS) is called a subdominant ultra-metric space relative to the metric space (X, d).

#### Calculation method

As proposed by Engle [21], we utilize the DCC-MV-GARCH model to figure out dynamic conditional correlation coefficients and distances among indexes. We define the return of stock *i* on date *t* as follows:

$$S_{i}(t)=lnP_{i}(t)\;-\;lnP_{i}(t\;-\;1)$$ (1)

where *P*_*i*_*(t)* denotes the closing price of stock *i* on date *t*.

To calculate the dynamic conditional correlations, we have to estimate the residuals exerting no autocorrelation for every stock returns series. The mean formulation for each series is obtained from an ARFIMAX model estimated in two stages with a general form as following:

$$S_{t}=\sum_{w=1}^{5}\beta_{w}DU_{w}+z_{t}$$ (2)

$$(1\;-\;\sum_{i=1}^{p}f_{i}L^{i}){\left(1\;-\;L\right)}^{d}\;z_{t}=(1+\sum_{j=1}^{q}\theta_{j}L^{j})\varepsilon_{t}$$ (3)

Where in the first estimation stage, *DU*_*w*_ denote dummy variables trapping potential day-of-the-week effects, *β*_*w*_ denote the corresponding regression coefficients, *z*_*t*_ represent the error terms. In the second estimation stage, first of all, we set the value of difference parameter *d* as 0 and the maximum of *p* and *q* to be 8. Herein, *φ*_*i*_ and *θ*_*j*_ denote ARMAX model coefficients, *L* is the lag operator, *ε*_*t*_ represent the residuals. We assume each residual obtained from the former procedure as:

$$r_{t}/\Omega_{t-1}∼N(0,H_{t})$$ (4)

$$H_{t}\equiv G_{t}C_{t}G_{t}$$ (5)

Where *H*_*t*_ is a decomposed conditional variance–covariance matrix, and *G*_*t*_ is a diagonal matrix of time-varying standard deviations from univariate GARCH models. The general Gaussian–GARCH constraints, i.e. non-negativity and stationarity were exerted. *C*_*t*_ is the time-varying conditional correlation matrix with the elements on the diagonal equal to unity:

$$C_{t}=diag{\left\{Q_{t}\right\}}^{-1}Q_{\;t}diag{\left\{Q_{t}\right\}}^{-1}$$ (6)

$$Q_{t}=\left(1\;-\;\sum_{p=1}^{P}\alpha_{p}\;-\;\sum_{q=1}^{Q}\beta_{q}\right)\bar{Q}+\sum_{p=1}^{P}\alpha_{p}\left(s_{t\;-\;p}s_{t\;-\;p}^{T}\right)+\sum_{q=1}^{Q}\beta_{q}Q_{t\;-\;q}$$ (7)

where *s*_*t*_ are standardized residuals, $\bar{Q}$ is the matrix of unconditional correlation of *s*_*t*_. The estimation of parameters *α*_*p*_ and *β*_*q*_ are performed by quasi-maximum likelihood based on adding up the quasi-likelihood functions of subsets of assets. Then the correlation coefficient between index *i* and *j* is obtained from the following formula:

$$\rho_{t,i,j}=\frac{q_{t,i,j}}{\sqrt{q_{t,i,i}q_{t,j,j}}},\;i,j=1,2,\ldots ,n;\;i\neq j$$ (8)

Nevertheless, the estimation of DCC within a single system is expected to generate biased results. Accordingly, we employ the individual bivariate DCC approach by Hafner and Reznikova [28] to calculate all of the bivariate DCCs and subsequently obtain the N×N correlation matrices C^DCC^t. After all the procedures, we transform the correlation coefficients to distance metrics between each pair of CSI industry indices as in Mategna [27]:

$$d_{{}_{ij}}^{t}=\sqrt{2(1\;-\;\rho_{{}_{ij}}^{t})}$$ (9)

The formula fulfills the requirements of distance. The N×N distance matrix is used to determine the minimum spanning tree (MST) which is constructed using Kruskal’s algorithm and presented in section 3.1.

Furthermore, we also calculate the rolling correlation coefficients and distances among indexes to verify the merits of networks constructed based on rolling correlation coefficient (RC) technique and DCC approach. The rolling correlation coefficient between index *i* and *j* is defined as:

$$\rho_{{}_{ij}}^{t\prime }=\frac{\langle S_{{}_{i}}^{t}S_{{}_{j}}^{t}\rangle -\langle S_{{}_{i}}^{t}\rangle \langle S_{{}_{j}}^{t}\rangle }{\sqrt{\left[\langle S_{{}_{i}}^{t}{}^{2}\rangle -{\langle S_{{}_{i}}^{t}\rangle }^{2}][\langle S_{{}_{j}}^{t}{}^{2}\rangle -{\langle S_{{}_{j}}^{t}\rangle }^{2}\right]}}$$ (10)

where the notation 〈⋯〉 indicates a time average over the trading days included in the return vectors *S*_*i*_. Likewise, we transform the rolling correlation coefficients to distance between two indexes, given by:

$$d_{{}_{ij}}^{t\prime }=\sqrt{2(1\;-\;\rho_{{}_{ij}}^{t\prime })}$$ (11)

### Centrality

Centrality represents the position of points in a network [29]. Accordingly, we can utilize the centrality estimation to identify the core nodes in stock network. In this study, we propose an explicit analysis on the time-varying MST using three different quantitative definitions of centrality as follows:

Degree centrality is defined as the number of adjacent edges involved with a given node [26]. This estimate can be written in terms of:

$$D(i)=\frac{1}{N\;-\;1}\sum_{i\neq j}g^{t}{}_{ij}$$ (12)

where *g* represents the connections between node *i* and *j*. To illustrate, a higher degree centrality value is associated with a greater number of links for a stock.

Betweenness centrality is defined as the number of geodesics (shortest paths) going through a vertex or an edge, which is a measure of one node’s importance as an intermediate element between other nodes in the network [30]. It is obtained from the following formula:

$$B(k)=\sum_{i,j}\frac{n_{ij}(k)}{m_{ij}}$$ (13)

Where *n*_*ij*_*(k)* represents the number of shortest geodesic paths traversing *k* between nodes *i* and *j*, while *m*_*ij*_ denotes the aggregate number of shortest geodesic paths between node *i* and *j*.

Closeness centrality gauges how many steps is required to access every other vertex from a given vertex whose value is high for strongly connected central nodes and vice versa[30]. This measure has been defined as follows (we define closeness centrality to be the inverse of the total distance so that higher values indicate greater centrality herein):

$$C(i)=\frac{1}{\sum_{j=1}^{N}d(i,j)}$$ (14)

where *d(i*, *j)* indicates the minimum path distance between node *i* and *j*. Generally, stock possesses larger value of centrality is also accompanied with greater linkage effect exerting on other stocks in the network.

### Network topological properties

We adopt several evaluative criteria to capture the topological and statistical natures of stock networks. We calculate the mean of correlation coefficients, the mean of distances, as well as the normalized tree length to exploit the interdependence relations and dynamic linkage effects in the analyzed stock networks. The mean correlation coefficients are defined as follows:

$$\bar{\rho }=\frac{1}{N(N\;-\;1)}\sum_{i\neq j}\rho^{t}{}_{ij}=\frac{2}{N(N\;-\;1)}\sum_{i<j}\rho^{t}{}_{ij}$$ (15)

The mean of distances in distance matrix can be written as:

$$\bar{d}=\frac{1}{N(N\;-\;1)}\sum_{i\neq j}d^{t}{}_{ij}=\frac{2}{N(N\;-\;1)}\sum_{i<j}d^{t}{}_{ij}$$ (16)

The normalized tree length (NTL) is proposed by [31], which is utilized to analyze the linkage effects among different currencies, and is obtained from the following formula:

$$NLT(t)=\frac{1}{N\;-\;1}\sum_{d_{ij}\in D}d^{t}{}_{ij}$$ (17)

where is the set of edges in MST.

## Results and Discussions

### Overall network structure analysis

**Fig 1:**
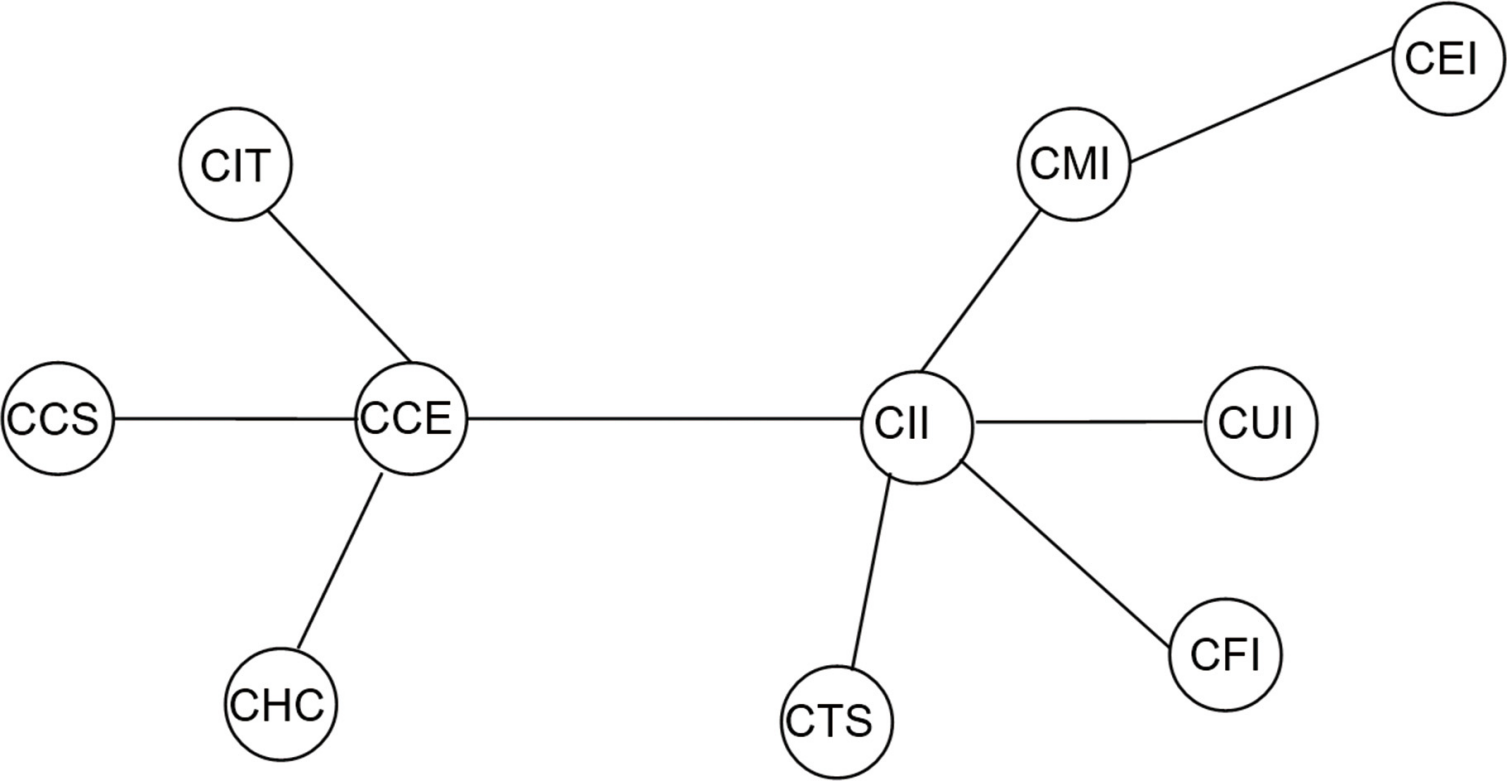
Minimum spanning tree of one-tier CSI industry indices. (2002/1/6-2015/7/1).

In this section, we have investigated non-linear comovements that arise with the stock indexes based on dynamic conditional correlations (DCC) of the daily CSI industry indices during three different time intervals. We have used two approaches, namely the MST and HT method as a means to trace and analyze the statistical features, hierarchical properties and dynamical linkages among Chinese stock market over the abovementioned study periods. The MSTs of indices in the stock market based on DCC finds a subset of the edges forming a tree that includes every vertex, where the total weight of all the edges in the tree is minimized. After obtaining the stocks minimum spanning tree, we can map the subdominant ultra-metric space into corresponding hierarchical trees, and thereby understanding the structure distribution and engaged linkages within the national stock clusters further. The horizon and vertical axes represent the worldwide industry code and the ultrametric distance at which the two industries are combined respectively. Finally, we have taken the MSTs of one-tier CSI industry indices based on rolling correlation coefficient (RC) technique for examples to verify the limitations of RC technique for case of high frequency financial data. The main conclusions of the topological properties of the stock networks consist of the following:

(1) From Fig 1 which depicts the structural information of one-tier C SI industry indices, we can capture several important features. We observe two clusters with CII and CCE at their centers respectively, which consistently match with their general production activities or their strong connections. The former is made up of a set of indexes that are strongly correlated with each other, namely CII, CMI, CUI, CEI, CFI and CTS. CII maintains a predominant position with greater degree than the other nodes. This cluster is denominated as the “Industrials” cluster for a majority of industries of the six indexes are classed as the secondary sector. Furthermore, the latter is composed of CCE, CIT, CHC and CCS such that CCE at its core. Of interest in this group is that all industries of the internal indexes are identified as consumer-exposed, hence we name it as the “Consumers” cluster exactly. One can see that the indices in the recent Chinese market’s MST are distinctly assembled in line with their industry categories, with the most probable interpretation for this phenomenon being that the frequent economic activities and economic interactions between related industries in the real economy system, thus leading to more tightly connected indices and more intense comovements among them in the stock market, which reflect in the decreasing average path length of the MST. This discovery provides a strong practical evidence for the connection between the structural linkages and the real national stock markets.

**Fig 2:**
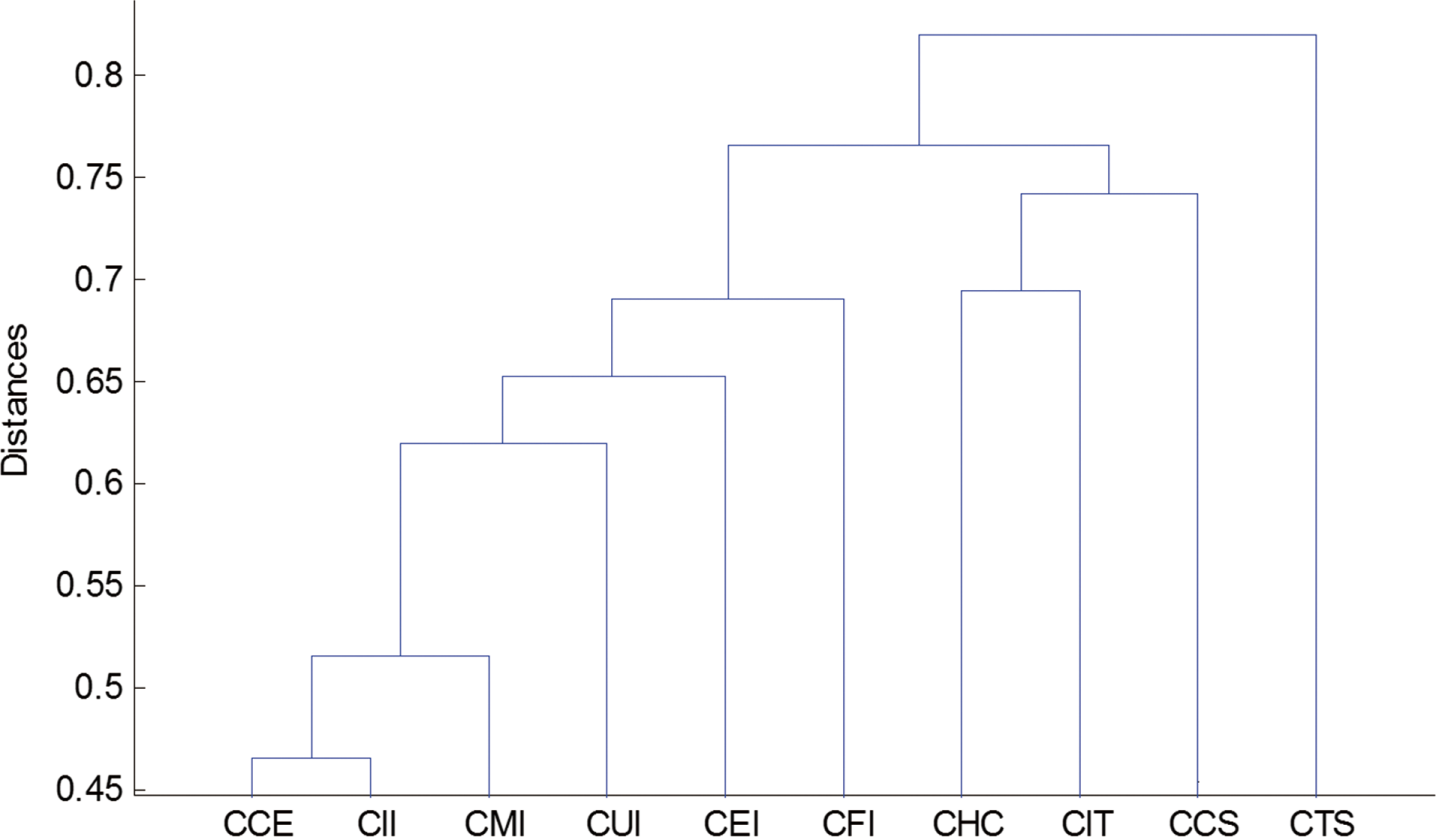
Hierarchical tree of one-tier CSI industry indices. (2002/1/6-2015/7/1).

The HT obtained starting from the MST described in Fig 1 is illustrated in Fig 2. In this figure, one can see that the distance between CII and CMI is the smallest of the sample, indicating a strong relationship between these two industries. Not surprisingly, given the importance of its social role, the CUI is the next to join. CII, CMI, CUI, CEI, and CFI are very close and make up the cluster, meanwhile CHC, CIT, and CCS have been at the same level, implying an intense connection of these industries, as can also be seen in Fig 1A.CTS has been at a high layer in this period and distant from other industries, indicating that its linkage with others is extremely weak. Interestingly, CCE is isolated from the “Consumers” cluster and closely interrelates with the hub of “Industrials” cluster i.e. CII, which may be mostly ascribed to the magnitude discrepancy of linkage effects between the two indexes and within the “Consumers” cluster.

**Fig 3:**
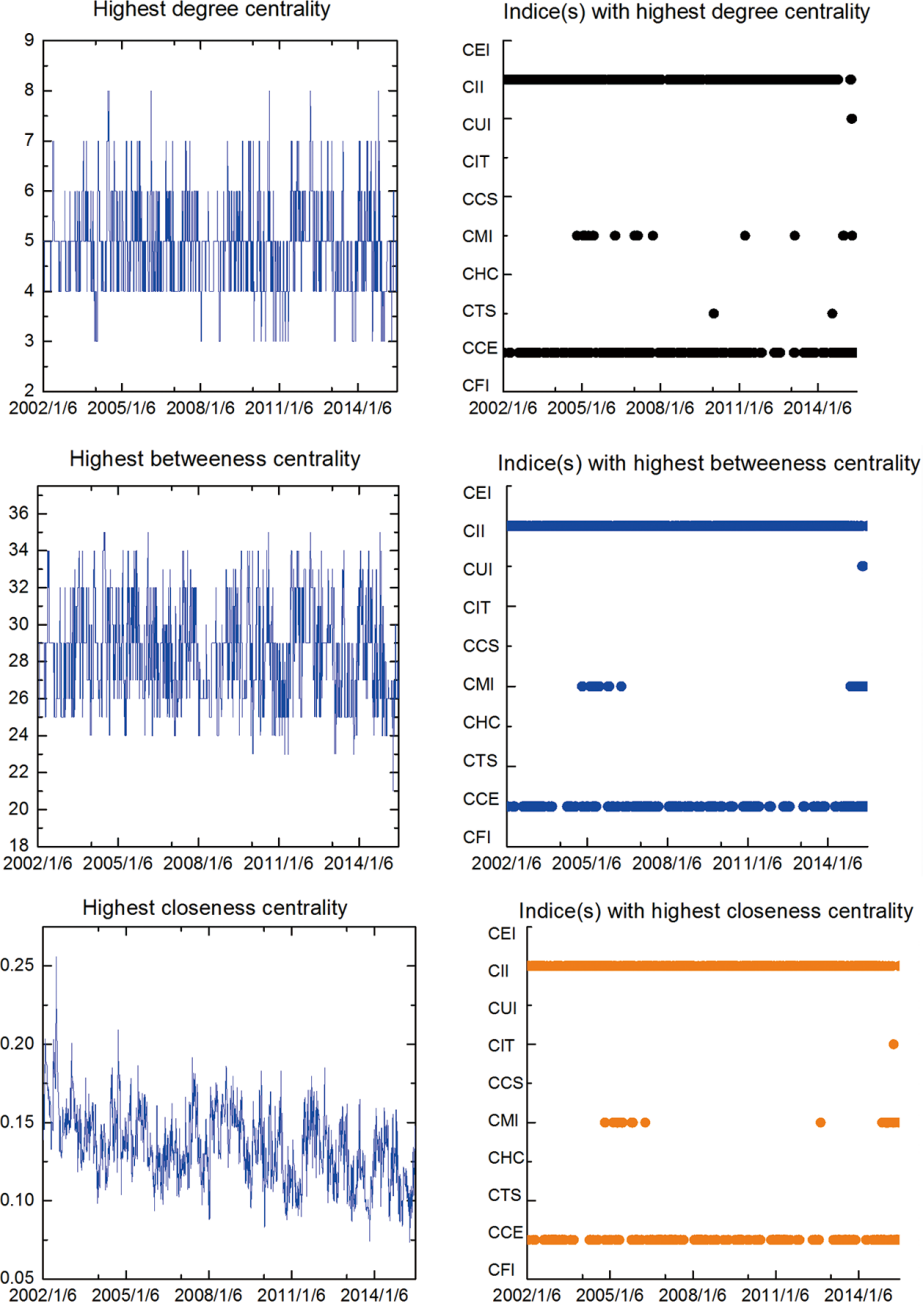
Time-varying highest centrality measures and the corresponding indices in the one-tier MST. (2002/1/6-2015/7/1).

In order to illustrate the robustness of the network interdependence results related to one-tier CSI industry indices, we propose three kinds of ‘Centrality’, namely, the degree centrality, betweenness centrality and closeness centrality as implementable criterions for selecting the intrinsic key vertex. In particular, a larger centrality value is related to a more influential position for a stock index within the system. The time-varying highest centrality measures and the corresponding indices based on the MST displayed in Fig 1 are plotted in Fig 3. Considering the numerical variation tendency of all kinds of centrality values, CCE and CII maintain an overwhelming advantage for almost all the time, showing that they keep highly correlated to other indices and exerting pronounced influences on other elements from dynamic perspective. In consequence, they are virtually the unquestionable most essential vertices in the stock network during all the time period, which provides more compelling evidence in support of the applicability of the aforementioned analyses and pinpoints the key roles of the two industry indices. In addition, it is observed that the highest degree centrality periodically floated up and down around the range (3, 8), and the highest betweenness fluctuated within a narrow band (between 24 and 34), while the highest closeness centrality value consistently manifest a moderate decreasing pattern during the time interval.

**Fig 4:**
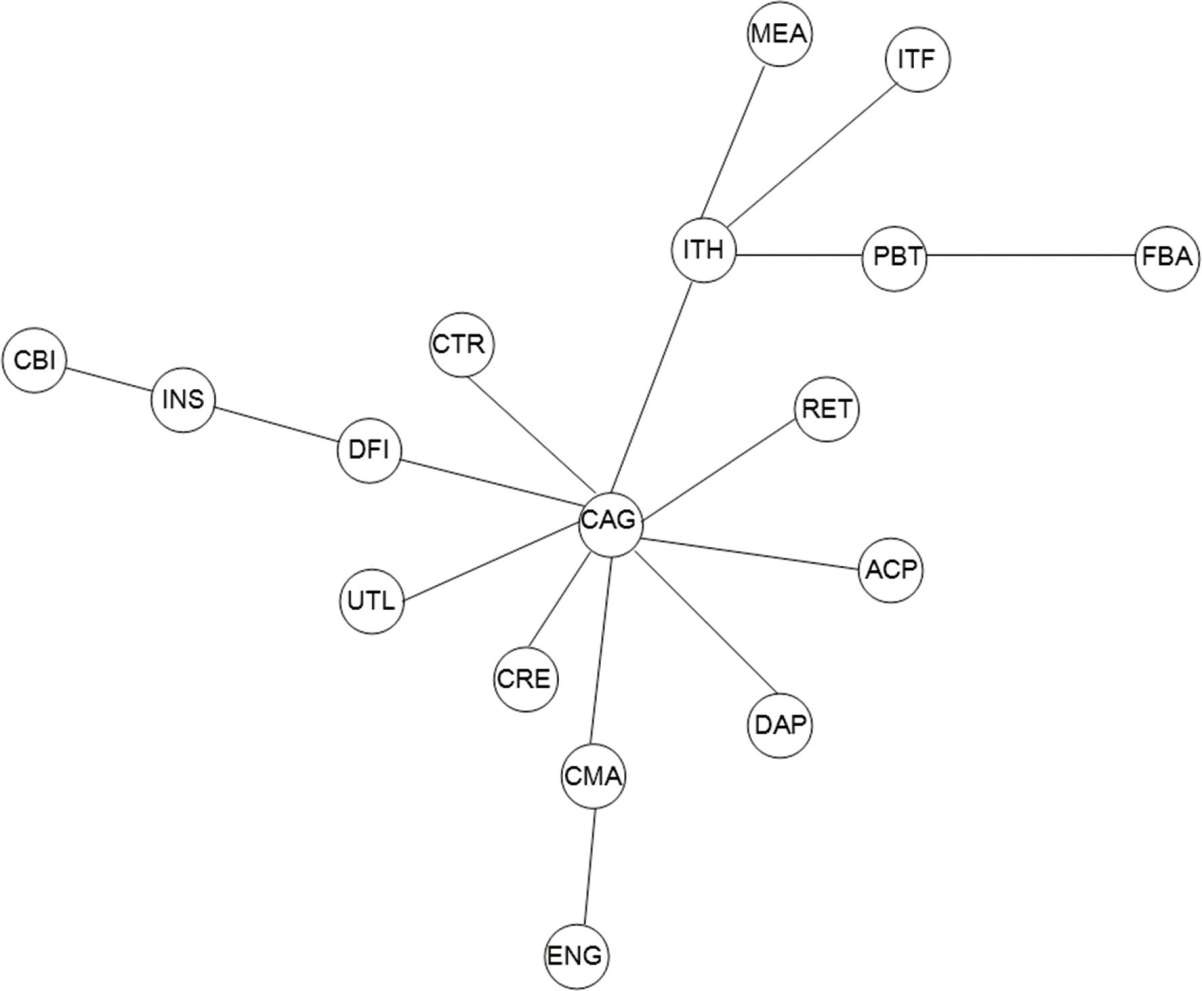
Minimum spanning tree of two-tier CSI industry indices. (2009/1/6-2015/7/1).

(2) As previously mentioned, CII and CCE has long been at the central location in China’s stock market in the MST of one-tier CSI industry indices (hereafter referred to as “one-tier MST”), while in the other two networks, things are different. For instance, as portrayed in Fig 4, two obvious stock clusters are observed, namely the “Capital Goods” cluster and the “Internet” cluster with CAG and ITH as the central nodes respectively. Compared to the stock clusters with CCE and CII at their centers in Fig 1, it is obvious that the industry attributes of the two central nodes have changed substantially in the latter case. The former consists of multiple indexes directly from industries composed of Retail, Durable & Apparel, Auto & Component, Materials, Real Estate, Utilities, Diversified Financials and Trans, and indirectly from industries including Bank, Insurance and Energy. However, the latter is composed of numerous indices all related to Information Technology, although there are two indices having diverse classifications namely Pharma & Biotech and Food & Beverage, which demonstrates observably strong industrial features. In a word, CAG and ITH are confirmed as two dominating indices in the Chinese equity market and maintain significant roles in the MST. Specifically, compared to the stock clusters in Fig 1 whose degrees of central nodes (CCE and CII) are 4 and 5, respectively, apparent changes occurred in the Chinese stock market during the second time interval. The degrees of CAG and ITH in two-tier MST are 7 and 4, respectively, providing strong proof of tighter interdependence among stock network. Additionally, it is interesting to discover that these clusters are still homogeneous in regard to the economic relations of the industries as is the case in Fig 1.Generally, companies endowed with the same category are highly related to each other in comparison to companies of different categories, providing a strong evidence that stocks can be clustered in accordance with their industrial categories taxonomy due to the close interdependence among economic agents in the nationwide arena.

**Fig 5:**
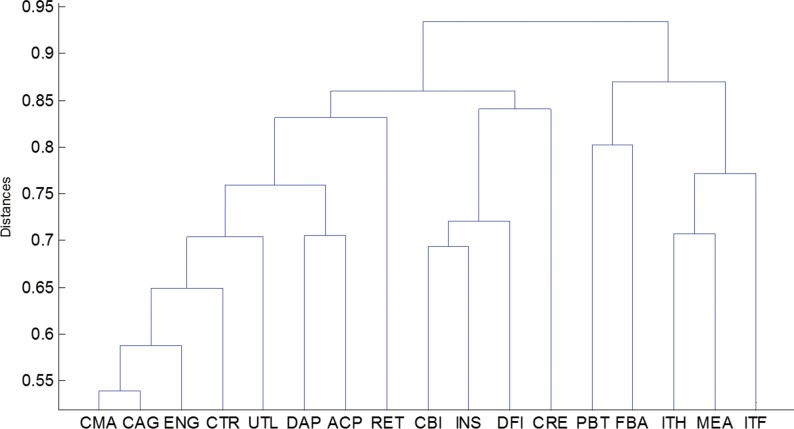
Hierarchical tree of two-tier CSI industry indices. (2009/1/6-2015/7/1).

The HT which corresponds to Fig 4 is shown in Fig 5. Given that the magnitude of distances among group of industries denote an adverse correlation degrees, we can rank them as follows: the first cluster, the more prominent and strongly connected cluster, is the cluster of CAG,CMA,CTR,UTL, DAP, ACP, RET, and ENG with the smallest distances between them; subsequently, PBT, FBA, ITH, MEA and ITF of the “Internet” group with greater distance between them, suggesting a weaker linkage effect among them. Intense relation of the “capital” group are mostly ascribed to the economic liaisons namely the trading connection among these industries.

**Fig 6:**
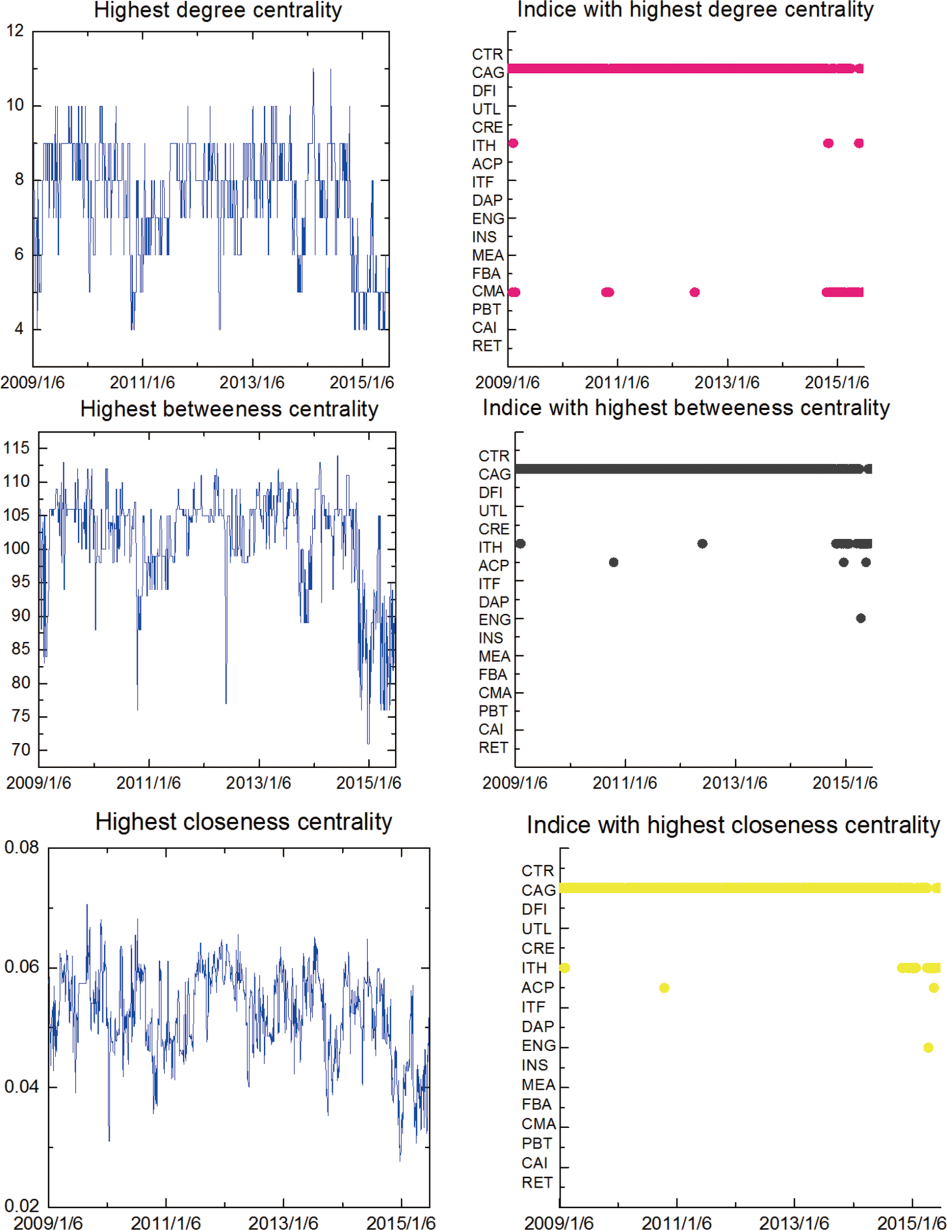
Time-varying highest centrality measures and the corresponding indices in the two-tier MST. (2009/1/6-2015/7/1).

**Fig 7:**
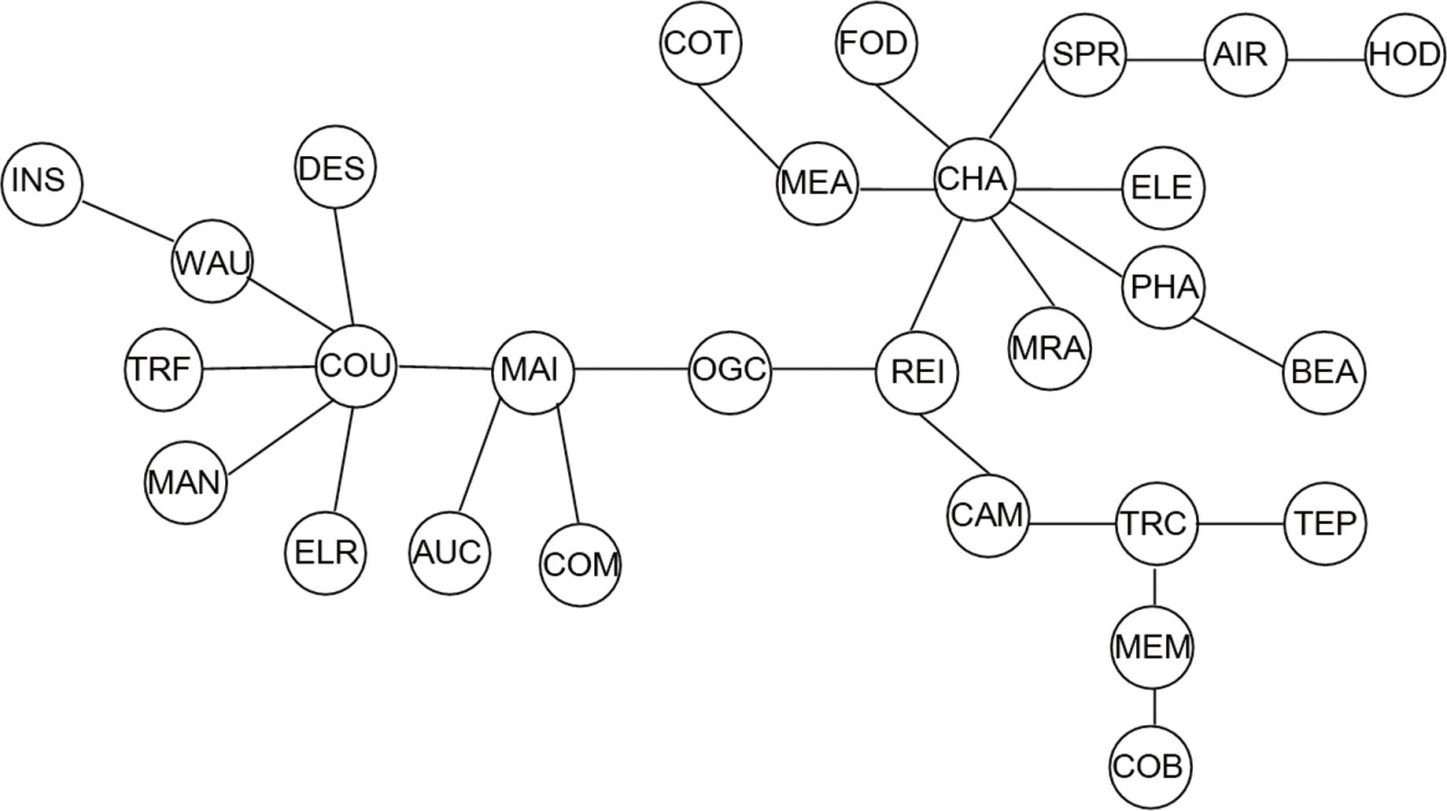
Minimum spanning tree of three-tier CSI industry indices. (2009/1/6-2015/6/25).

The time-varying highest centrality measures and the corresponding indices based on the MST displayed in Fig 4 are illustrated in Fig 6.The graphs consistently show the fact that the CAG vertice takes the highest centrality values for almost all the time (sometimes the ITH). Hence, it, no doubt, plays the most important role in the stock network during the study period, thereby representing more assertive evidence for the high robustness of the aforementioned exploratory findings underlying comovements across local stocks related to two-tier CSI industry indices. In addition, it is observed that the highest degree centrality periodically floated up and down around the range (4, 11), similarly, the highest betweenness and closeness centrality values fluctuated within the bands (71,114) and (0.03, 0.07) respectively.

(3)From Fig 7 which demonstrates the structure information of three-tier CSI industry indices, we can discover several features which are widely divergent from the former cases as follows. Three remarkable stock industry index clusters are observed in this time interval, namely the “Construction” cluster, the “Household” cluster and the “Property related” cluster with COU, CHA and REI at their centers respectively. As one can see, significant changes have taken place in the industry attributes of the central stock indices in three-tier MST when compared with the former cases. The first group is denominated as the “Construction” cluster for the reason that all industries of the indexes are recognized as members associated with construction trades, except for INS, which suggests the importance of building industry in the entire national stage. The second group comprises multiple indexes directly from industries composed of Media, Food, Specialty Retail, Electrical Equip, Pharm and Multi Retail, and indirectly from industries including Computers, Airlines, Beverages and Household Durables. The last group consisted REI, CAM, TRC, MEM, TEP and COB, all of which originate from industries related to the real estate business. It worth noting that both COU and REI maintain central positions in the network structure, which is in parallel to the critically essential roles of infrastructure construction and real estate in the Chinese society, thus underpinning their importance in the MST. The degrees of COU, CHA and REI are 6, 7 and 4 respectively which is diverse from the aforementioned two MSTs, indicating that the three-tier stock network got more dispersed on the whole but stocks connect more tightly while the comovements among them are more intense on smaller scales than the rest, which is manifested intuitively in Figs 1, 4 and 7. Interestingly, stock market indexes’ synchronization to their industry attributes is also observed in this section. To elaborate, there appears to be tighter connections and more observably strong clustering among the indexes situated in the same industry category. That is to say, stocks are inclined to connect with their peers within relevant industries, as is the case in aforementioned MSTs.

**Fig 8:**
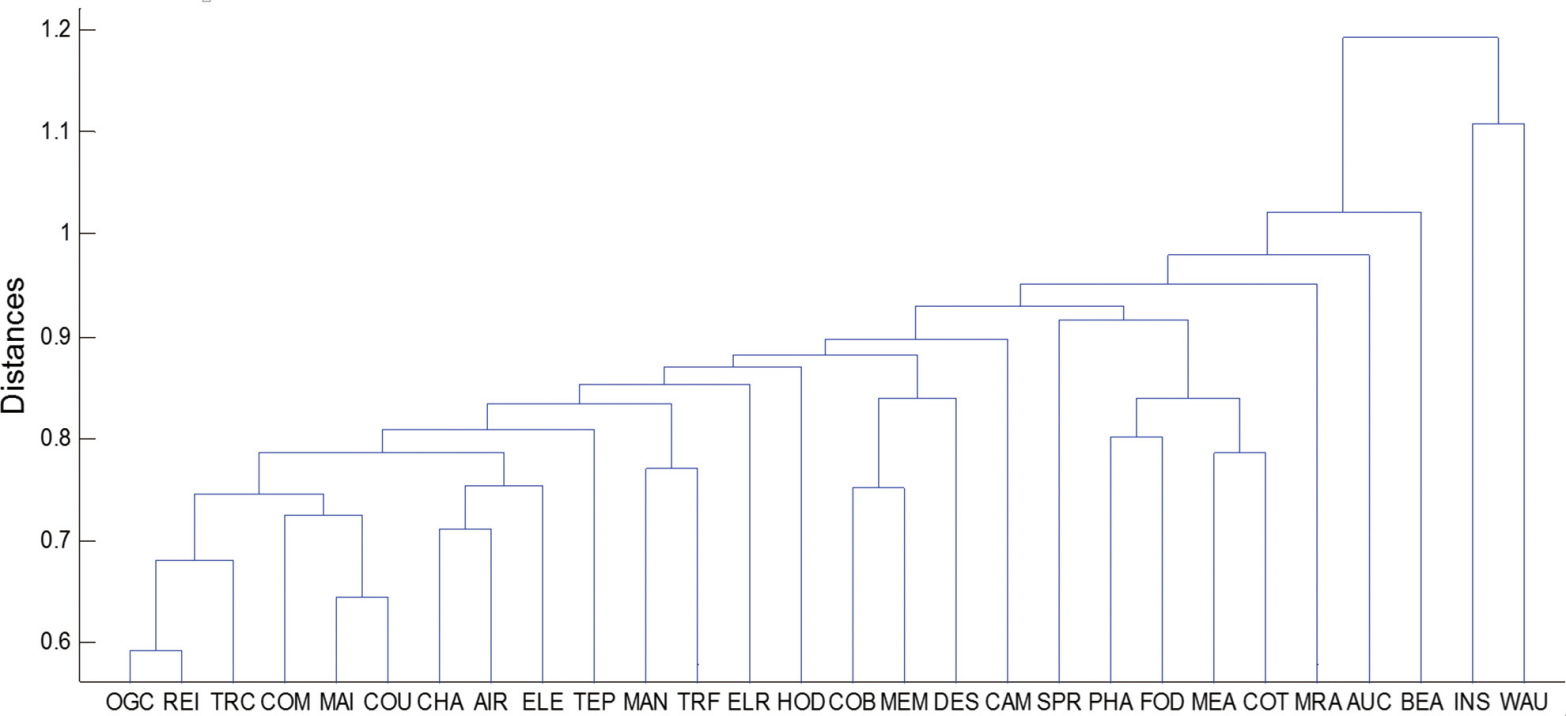
Hierarchical tree of three-tier CSI industry indices. (2009/1/6-2015/6/25).

The HT of the subdominant ultra-metric associated to the MST is shown in Fig 8. The existence of three clusters are clearly identified. With respect to the correlation degrees, the “Construction” group maintains the first place, as OGC, REI, TRC, COM, MAI and COU have still been at the same level, implying a close proximity of these industries. Next follow the “Household” group which is composed of CHA, AIR and ELE, with CHA playing a central role in this local network, indicating a strong linkage among them. And in third place are SPR, PHA, FOD, MEA, COT and MRA which show greater distance between their nodes than the first two groups. INS and WAU have been at a high layer in this period and distant from other industries, indicating that their linkages with others are especially weak.

**Fig 9:**
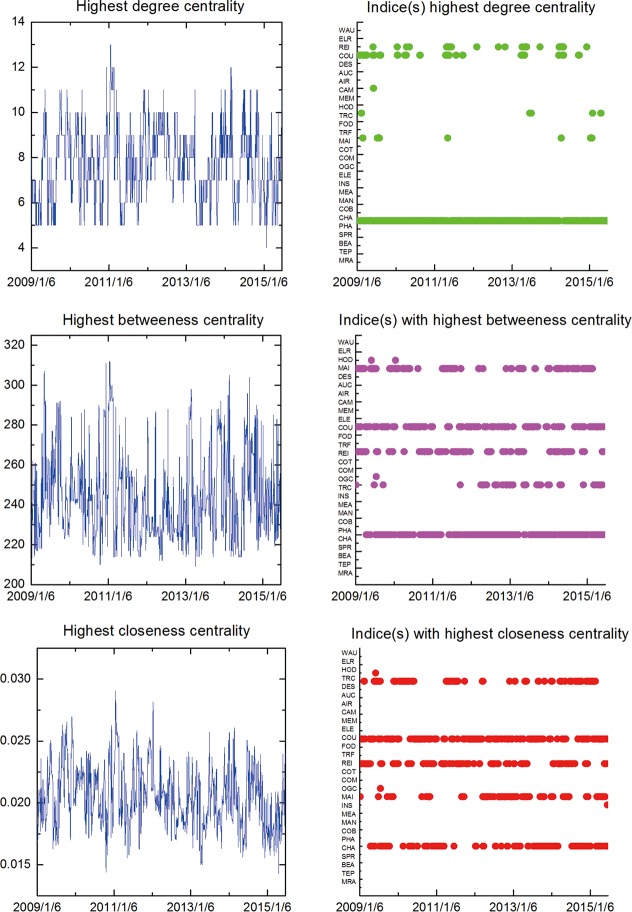
Time-varying highest centrality measures and the corresponding indices in the three-tier MST. (2009/1/6-2015/6/25).

The time-varying highest centrality measures and the corresponding indices based on the MST displayed in Fig 7 are demonstrated in Fig 9. According to the new case, it is noteworthy that the unique winner does not exist associated with the numerical centrality values. To specify, the COU, CHA and REI vertices (listed in their pecking order) take the top three places in terms of the centrality values of all kinds, which effectively clarifies their essential positions among all the elements in the stock network and, as well, supplies strong evidence for the credibility of the aforementioned conclusions concerning the linkage effects between stock market. More precisely, it is observed that the highest degree centrality values periodically floated up and down around the range (5, 11), likewise, the highest betweenness and closeness centrality values fluctuated within the bands (210,300) and (0.015,0.028) respectively.

**Fig 10:**
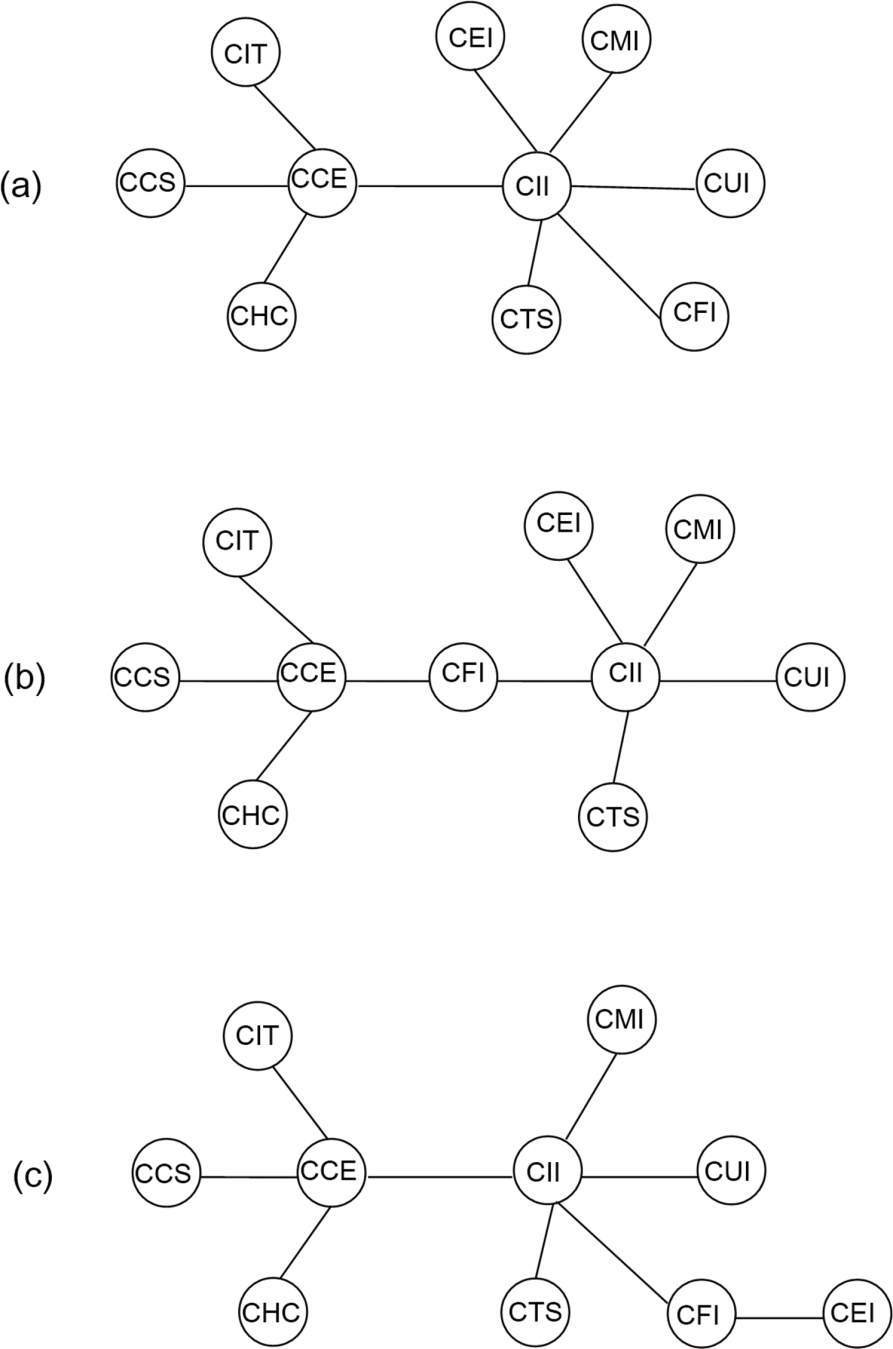
MST-RC of one-tier CSI industry indices. (a) shows the MST-RC of one-tier CSI industry indices during the whole study period (2002/1/6-2015/7/1). (b) shows the MST-RC of one-tier CSI industry indices on October 2008 (Window length = 6 months). (c) shows the MST-RC of one-tier CSI industry indices on October 2008 (Window length = 12 months).

(4) Furthermore, we construct networks based on the rolling correlation coefficients to dig into the differences between RC technique and DCC approach. Confined by space, we hereon take the one-tier CSI industry indices as a representative to plot topological pictures of sample indexes. Fig 10A. presents the MST-RC of three-tier CSI industry indices for the entire period as an overall picture. Fig 10B. and 10C illustrate the MST-RCs of three-tier CSI industry indices on October 2008, as an example of the period during US financial crises at different time windows (T = 6 months and T = 12 months, respectively).

Fig 4A is similar to Fig 1, but following differences have been observed. (i) It can be seen that the branch clusterization with CII and CCE at their centers respectively remained, but the tree structure changed since CEI is directly connected with CII. (ii) There is a significant increment on the linkage effects among the stock market, which is reflected in the reduced distance of links of two central nodes. With respect to the stock cluster with CCE at the center, its distances with the linked indices are 0.82, 0.92, 0.95, and 1.02, respectively in Fig 1, while in Fig 4A the corresponding distances decrease to 0.42, 0.53, 0.57, and 0.63, respectively. In the case of the stock cluster with CII at the center, its distances with the original linked indices (exclude CEI) are 1.02, 0.94, 0.82, 0.81, and 0.72, respectively in Fig 1, while in Fig 10A the corresponding distances decrease to 0.63, 0.54, 0.45, 0.43, and 0.36, respectively. In other words, indices connect more tightly while the comovements among them are more intense in the stock market network based on RC technique, which provides evidence of upward bias caused by data heteroskedasticity of rolling correlation coefficient estimate.

In order to illustrate the robustness of the results related to rolling coefficients, we plot the MST-RCs of one-tier CSI industry indices on October 2008, as a representative of the period during US financial crises at two time windows (Fig 10B and 10C). It can be clearly seen that, the worldwide spread of American subprime crisis undoubtedly contributes to an increase of the linkage effect of CFI. Besides, one interesting observation is noteworthy that with different choices of time window length, the MST structure has changed to some extent as well, which is a more assertive evidence for the higher robustness of results based on DCC than that based on rolling window method.

In conclusion, we interpreted the topology properties of the Chinese stock market adopting the minimum spanning tree analysis and hierarchical tree structure technique based on DCC estimation. It excludes the possibility of heteroskedasticity bias (correlations incline to be biased when volatility increases) and retains the ability to capture short term dynamics of the network structure, which is more applicable for the observation of daily changes in the temporal condition of the stock market. Although the structure natures are diverse from each other, the three analyses consistently yield findings in line with the status quo of the real economy in China during the study periods. Besides, the results for MSTs based on DCC estimation tend to be homogeneous which indicates that the central vertices are not random and are less sensitive to the subjective choice of estimation window, and the central vertices generally conforms to the expected results according to practical reasoning, which contradicts to the previous studies employing the rolling correlation coefficient (RC) process method [10, 11]. They provide evident proof of network clusters’ homogeneous tendency in regard to the economic communications of the industries in reality. In other words, a stock index is clustered on basis of the features of the sectors to which it belongs, thus building the interconnection between intuitive network topology structure information and virtual economic situation of financial markets. There appears to be greater clustering effect among the indexes belonging to related industrial sectors than those of diverse sectors. To specify, the one-tier industry indices can be divided into the “Industrials” cluster and the “Consumers” cluster, the two-tier industry indices can be divided into the “Capital Goods” cluster and the “Internet” cluster, and the three-tier industry indices can be divided into the “Construction” cluster, the “Household” cluster and the “Property related” cluster. It may greatly ascribe to the investors’ similar behavior modes applied to securities with relevant industry characteristics. In particular, CII and CCE, CAG and ITH as well as COU, CHA and REI have long been the central nodes in the networks they belong to, all of whose corresponding industries occupy significant positions in China’s development process of the three study sub-periods. The calculation of time-varying highest “centrality” tested the robustness of our results.

### Dynamic evolution analysis of network’s stability

To further dig into the stability of linkage effects, we utilize the network properties equations to obtain the mean correlation coefficient and mean distance, as well as the normalized tree length of these indices as indicators to illustrate the time series natures of connectivity structure information in a dynamic network. In particular, the normalized tree length and mean distance mean correlation coefficient are defined as the mean of the distances in the MST and Distance Matrices respectively, with higher values representing a tighter coupling effect among the indices and vice versa. Simultaneously, the mean correlation coefficient refers to the mean of coefficients in correlation matrix and is also related to the interdependence within the system. Conversely, the higher the value of the mean correlation coefficient is, the tighter the interconnections among the indices.

**Fig 11:**
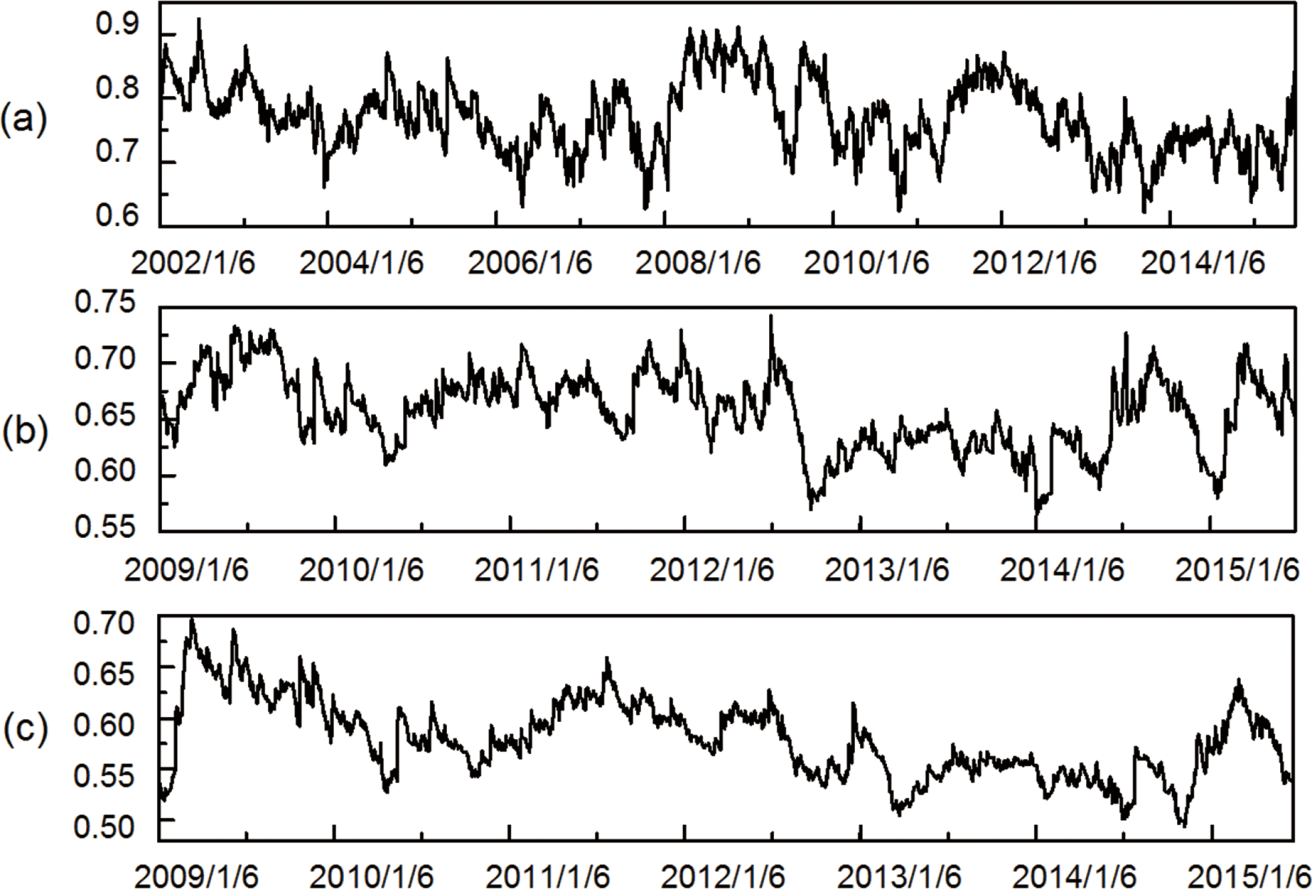
Dynamic mean correlation coefficients. (a) shows the one-tier MST (2002/1/6-2015/7/1). (b) shows the two-tier MST (2009/1/6-2015/7/1). (c) shows the three-tier MST (2009/1/6-2015/6/25).

Fig 11 presents the dynamics of mean correlation coefficient between all industry indices throughout the entire study periods with respect to the Chinese stock market. It is noteworthy that the mean correlation coefficient associated with the three stock networks periodically floated up and down in normal times but dramatically increased as reactions to U.S. debt-ceiling crisis (late 2008 to mid-2009) and European debt crisis (early 2011 to mid-2012), although with some delay, which arises a noticeable question concerning whether the dynamic market inter-correlations could be interpreted by the impact of international financial crises. In the case of European debt crisis, the mean correlation coefficient sharply rose and declined passing through the time interval. In the case of U.S. subprime crisis, the mean correlation coefficient climbed up to higher position in comparison with the European debt crisis, indicating a stronger interdependence relationship among stock indices. Between the two time breakpoints, the mean correlation coefficient remains anchored to a relatively lowered level. It undoubtedly verifies the conjecture that the numerical mean correlation coefficient variation may partly originate from the contagion effect of influential financial crises.

**Fig 12:**
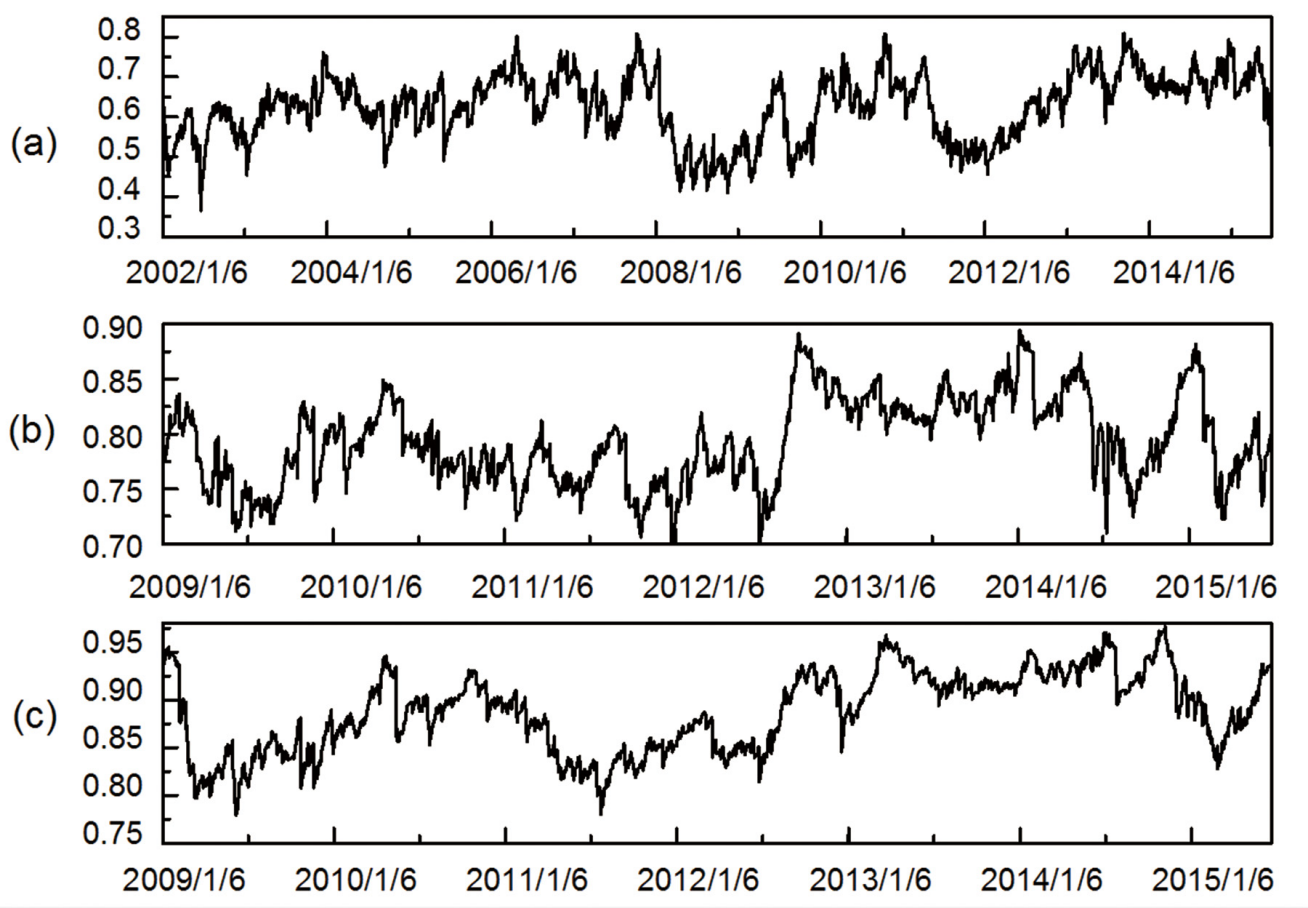
Dynamic mean distances. (a) shows the one-tier MST (2002/1/6-2015/7/1). (b) shows the two-tier MST (2009/1/6-2015/7/1). (c) shows the three-tier MST (2009/1/6-2015/6/25).

**Fig 13:**
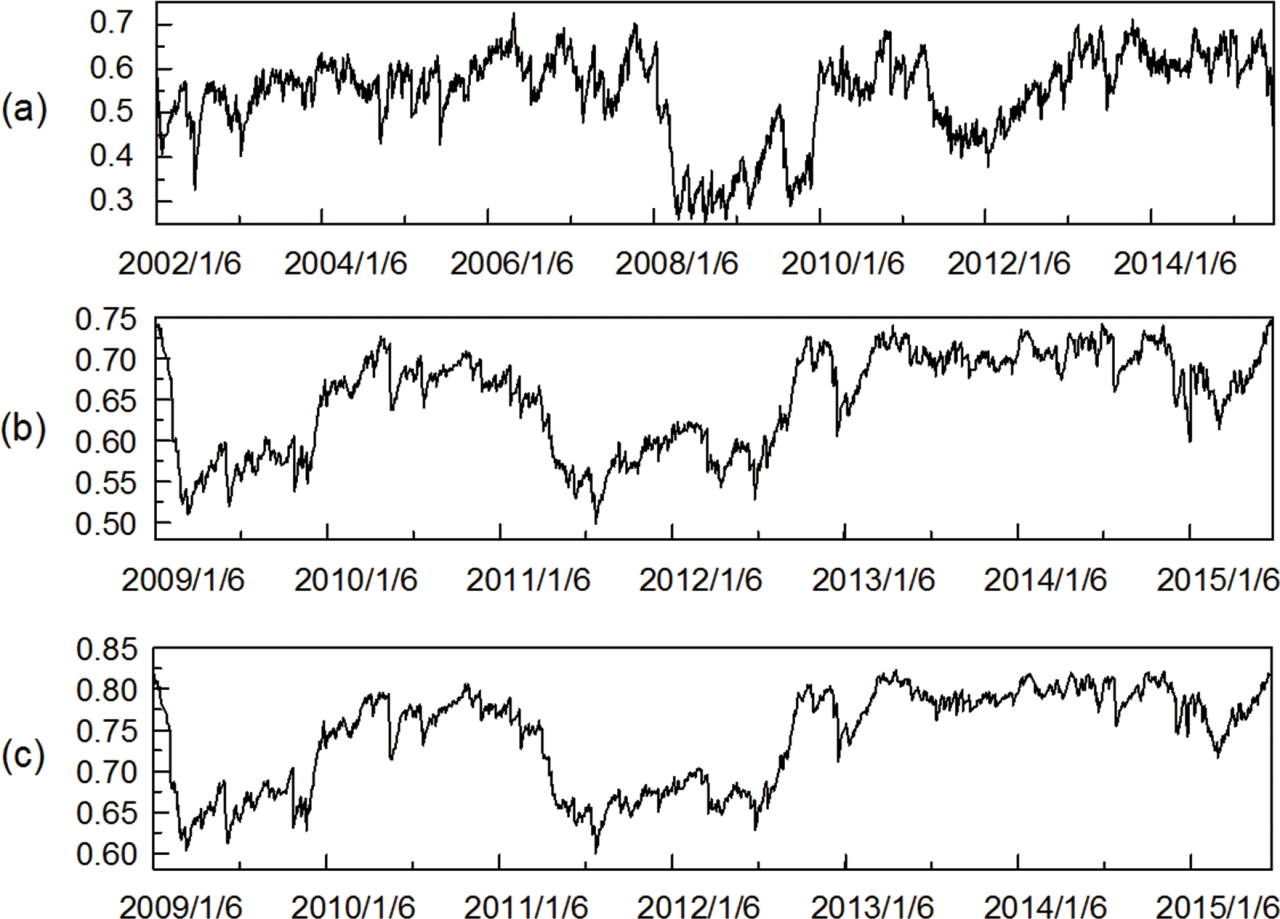
Dynamic normalized tree length. (a) shows the one-tier MST (2002/1/6-2015/7/1). (b) shows the two-tier MST (2009/1/6-2015/7/1). (c) shows the three-tier MST (2009/1/6-2015/6/25).

Figs 12 and 13 presented the mean distance and normalized tree length among all industry indices with respect to the Chinese stock market. Interestingly, we observed that the mean distance and the normalized tree length tend to follow similar fluctuation pattern. Specifically, both mean distance and normalized tree length demonstrate special movements around U.S. subprime crisis and European debt crisis, as often as is typically found for mean correlation coefficient. The mean distance of three stock networks periodically dipping and heaving around the range (0.3, 0.8), (0.7, 0.9) and (0.75, 0.95), meanwhile the normalized tree length of them fluctuated in the interval of (0.3, 0.7), (0.5, 0.75) and (0.6, 0.85), respectively. In the case of the European debt crisis, the mean distance and the normalized tree length rapidly steeply ascended at the outset, and then gradually declined afterwards. In the case of U.S. subprime crisis, they decreased to relatively lower level compared with European debt crisis, manifesting a tighter interdependence among stock network. After the events, both the normalized tree length and mean distance slowly rebounded subsequently. In this respect, we reasonably conclude that the normalized tree length and mean distance can capture the tendency of financial crisis despite always having a delayed informative cue, which is in parallel to the prior findings, which argues that the inter-nodal comovements in stock market are significantly affected by influential financial events in the reality [32].

In conclusion, the linkage effects among equity markets in the study region consistently manifest an increasing pattern along with the outbreak of global financial turmoil and subsequently revert to normal status in ordinary times. This result also provides powerful evidence for the finding of Flavin et al. (2008), which states that the linkages between equity markets appear to be instable [32].

### Network effects on stock returns

To further investigate the effects of network structure natures on virtual stock returns, we utilize the closeness centrality equations to obtain the inter-industry closeness as an indicator to illustrate the connectivity structure of a dynamic network. We select stocks’ closeness centralities as the indicator for dynamic stock market network linkages for the reason that a larger closeness centrality value is related to a more structurally influential position for a stock index within the system since a vertex of high closeness centrality can easily reach or be reached by others, so that they can represent the degrees of stocks’ inherent correlation risks [33]. Subsequently, we explore in depth the relations between stock’s centrality and its corresponding future returns to verify the conjecture that the stock with the tightest linkage to its network has the largest expected return among the nodes. In order to tackle this issue, we adopt the generalized method of moments (GMM) model proposed by Blundell and Bond (1998), which allows for the endogeneity of explanatory variables [34]. It provides the linear specification of the return of stock with the following formula:

$$r_{i}(t)=\alpha +\beta C_{i}(t)+\gamma r_{i}(t\;-\;1)+\lambda r_{sh}(t)+\varepsilon_{i}(t)$$ (18)

Where the *r*_*i*_*(t)* is the return of indice *i* in date *t*, *α* is the constant, *C*_*i*_*(t)* denotes the centrality for industry *i* in the stock networks in date *t*, *r*_*sh*_*(t)* is the return ratio of the Shanghai and Shenzhen Stock Market in date *t*, and *ε*_*i*_*(t)* is all other influential factors.

The regression results of three stock market networks are presented in Table 2. In the case of the stock market network of one-tier CSI industry indices, the regression model is valid and accurate, as the results of Sargen test, AR(1) test, AR(2) test and R^2^ are 1.000, 0.0124, 0.3184 and 0.8731 respectively, which excludes the possibility of autocorrelation and poor fitness. Interestingly, both the indice’s centrality value and return ratio of the Shanghai and Shenzhen stock market exert significant positive effect on the indice’s expected return, whereas the effect of its previous return is less significant. Moreover, the coefficient for the variable centrality is 0.1853, indicating that a one-unit increase in an index’s closeness across indices results in a nearly 19-percentage-point increase in the stock’s expected returns, when other factors remain constant. This result indicates that a stock’s future returns increase as the connections between the stock and other stocks increase.

**Table 2:** Dynamic GMM-regression results.

Variable	One-tier network	Two-tier network	Three-tier network
**Constant**	-0.3146**	-0.1125*	-0.0065*
**Closeness Centrality**	0.1853**	0.1037**	0.0664***
**Return (−1)**	0.3605*	0.0429**	0.1163**
**Return**_**sh**_	0.6374***	0.8835***	0.5268***
**No. of obs.**	25820	21046	34580
**No. of indices**	10	17	28
**Sargen test**	1.0000	1.0000	1.0000
**AR(1) test**	0.0124	0.0001	0.0000
**AR(2) test**	0.3184	0.9432	0.2127
**R**^**2**^	0.8731	0.8526	0.8574

The Sargan test refers to the two-step estimation results. AR (1) and AR (2) are the tests of first and second order autocorrelation in the residuals.

*, **, *** indicate significance at the 10%, 5% and 1% levels, respectively.

Next, we turn to the estimation of the expected return associated to the centrality factor with respect to the two-tier CSI industry indices. The regression model is valid and with good accurace, as the results of Sargen test, AR(1) test, AR(2) test and R^2^ are 1.000, 0.0001, 0.9432 and 0.8526 respectively, which excludes the possibility of autocorrelation and poor fitness. Furthermore, the centrality of a stock has a significant effect on the stock’s expected returns in networks; thus, the inter-connections between stocks or the positions of stocks in networks determine stock returns. Compared with the prior regression, no substantial changes have taken place in both the sign and significance of regression coefficients in this step, manifesting that the stock’s centrality has stable forecasting ability on its future return and simultaneously the positive regression coefficient denotes a positive centrality price of risk.

Finally, we examine the predictive ability of stock inter-connections with regard to stock returns of the three-tier CSI industry indices. The regression model is valid and accurate, as the results of Sargen test, AR(1) test, AR(2) test and R^2^ are 1.000, 0.0000, 0.2127 and 0.8574 respectively, which excludes the possibility of autocorrelation and poor fitness. It should be noted that one indice’s centrality value and return ratio of Shanghai and Shenzhen stock market have significant positive effects on an index’s expected return, whereas, the effects of its previous return is less significant. In addition, the coefficient for the variable centrality is 0.0664, suggesting that when other factors are held constant, a one-unit upward change in an index’s centrality will account for almost 7-percentage-point unit augment in the stock’s expected returns.

In conclusion, expected returns consistently manifest an increasing pattern along with the centrality value of the entire stock market networks, which strong demonstrates the results’ robustness. This high stability apparently advances the applicability of regression results in policy making for the Chinese market as they tend to exhibit wide range reliability. According to the aforementioned findings, equity returns are significantly affected by the inter-nodal influence and the relation between node centrality and market return is pronouncedly positive. It undoubtedly provides powerful evidence that central or ‘exogenous’ stock that is highly correlated to other stocks and projects substantial influences on the propagation of fundamental shocks in the economy, will pronouncedly gain higher expected return. Consistent with the theoretical prediction, we find support of a positive centrality price of risk. In this respect, we reasonably conclude that centrality helps to motivate the value premium as a causality risk premium: the expected return of value stocks in excess of growth stocks is a centrality premium to some extent.

## Conclusion

The focal points of this study are to synthetically analyze comovements of Chinese equity market using the MST and hierarchical tree method, to further capture the time-varying stock network characteristics across different industries by estimating mean correlations and mean distances, as well as the normalized tree length of these indices in three stock networks during the study periods, and to examine the effects of network linkages on stock returns.

An important contribution in the network analyses is that we find indices of similar industry nature flock together, which provides evidence of the stock market indexes clustering behaviors’ synchronization to their industry properties, and confirms network as the portrait of the real economic circumstance theoretically and empirically. That is, there appears to be greater clustering effect among the indexes belonging to related industrial sectors than those of diverse sectors. The evidence implies that the Chinese equity market develops homogeneous clusters on the strength of the industry.

Moreover, the dynamic evolution investigation of network structure suggests that the network is relatively stable over the time. In particular, CII and CCE, CAG and ITH as well as COU, CHA and REI have long been the central nodes and are highly correlated to other indices in the three sub-networks, respectively, which may mostly ascribe to the important position of the corresponding industries in China’s development process. In this sense, they play critically essential roles in the stock networks and may consistently project substantial influences on the propagation of fundamental shocks in the economy. As a consequence, regulators and investors should keep in mind that the central nodes in stock network deserve particular attention as the ever-burning lamps of vast universe of equity market, no matter from the perspective of financial risk supervision or pursuit for earnings. Nevertheless, prominent changes have taken place in the network during two exceptional periods, i.e. the U.S. subprime crisis and European debt crisis. It is obviously noticed that the network’s interdependence relationship strengthened substantially, which suggests that the network comovements variation may partly originate from the contagion effect of influential financial crises in reality.

In addition, this research sheds light on the asset pricing mechanism of stock market. Though stock market networks have been extensively explored, we have extended this line of study to the effects of network topological properties on stock returns. Our regression results indicate that the dynamic correlation between centrality and stock market returns is consistently positive over time, thereby representing evidence that stock future returns are significantly affected by the extent of the interdependence for that stock in the concerned equity markets. In essence, the closeness for stock represents the degree of its inherent correlation risk [33]. To specify, the stock with most connections to its network obtains the largest expected return among the central nodes, while the stock most influenced by its ‘hub’ obtains larger risk premium among periphery nodes. From the perspective of psychological factors, stock market investors usually tend to present characteristics of risk aversion, and this tendency even magnifies during the periods of the financial crises. Consequently, it is reasonable to expect that investors require high returns for those assets posited at the center of the network structure as a premium for the magnified contagion risk. In this way, network co-movement plays a critically important role determining the asset pricing mechanism and deserves a positive risk price.

The findings are enlightening since few prior researches have focused on the network topologic metrics in the financial domain. From an economic perspective, our empirical estimation of comovements, linkages and stock returns conveys a host of inferences, i.e. the influence of one given node towards other indices, the risk attributes, the intrinsic return potential, thereby providing profound insights as to construct diversified portfolio or make risk management in terms of their topological location information in equity networks. For instance, investors can curb repetition of highly related assets when making portfolio allocations, and they can focus on the trends of the industry indices correspond to their holding assets when making investment decisions. In this way, the proposed method provides insightful implications that facilitate investors and regulators in analyzing stocks based on the co-moving industries and highlights that they should pay close attention to the “hub” nodes rather than monitor every node within the system.

## Supporting Information

S1 Text The Shanghai and Shenzhen stock market industry indice price. (RAR)

## References

[1] Nier E, Yang J, Yorulmazer T, Alentorn A. Network models and financial stability. Journal of Economic Dynamics and Control. 2007; 31(6): 2033–2060.

[2] Tse CK, Liu J, Lau FCM. A network perspective of the stock market. Journal of Empirical Finance. 2010; 17(4): 659–667.

[3] Chen TQ, He JM. A network model of credit risk contagion. Discrete Dynamics in Nature and Society. 2012;Article ID 513982, 13 pages. doi: 10.1155/2012/513982

[4] Qiao H, Li Y, Xia Y. Analysis of linkage effects among currency networks using REER data. Discrete Dynamics in Nature and Society. 2015;Article ID 641907, 9 pages. doi: 10.1155/2015/641907

[5] Eryigit M, Eryigit R. Network structure of cross-correlations among the world market indices. Physica A. 2009; 388: 3551–3562.

[6] Dewandaru G, Rizvi SA, Masih R, Mansur M, Alhabshi SO. Stock market co-movements: Islamic versus conventional equity indices with multi-timescales analysis. Economic Systems. 2014; 38:553–571.

[7] Eoma C, Oh G, Jung WS, Jeong H, Kimd S. Topological properties of stock networks based on minimal spanning tree and random matrix theory in financial time series. Physica A. 2009; 388:900–906.

[8] Maman AD, Gan SL. Minimal spanning tree problem in stock networks analysis: An efficient algorithm. Physica A. 2013; 392(9):2226–2234.

[9] Tumminello M, Aste T, Matteo TD, Mantegna RN. A tool for filtering information in complex systems. Proceedings of the National Academy of Sciences of the United States of America. 2005; 102(30):10421–10426. 16027373 10.1073/pnas.0500298102 PMC1180754

[10] Huang FX, Gu J, Li YX, Su JQ. Linkages and dynamic stability of the national of global primary stock index before and after the financial crisis. Systems Engineering—Theory and Practice. 2010; 10:1729–1740.

[11] Gilmore CG, Lucey BM, Boscia M. An ever-closer union? Examining the evolution of linkages of European equity markets via minimum spanning trees. Physica A. 2008; 387(25):6319–6329.

[12] Ulusoy T, Keskin M, Shirvani A, Deviren B, Kantar E, Dönmez CC. Complexity of major UK companies between 2006 and 2010: hierarchical structure method approach. Physica A. 2012; 391 (21): 5121–5131.

[13] Coelho R, Hutzler S, Repetowicz P, Richmond P. Sector analysis for a FTSE portfolio of stocks. Physica A. 2007; 373:615–626.

[14] Brida JG, Risso WA. Hierarchical structure of the German stock market. Expert System Application. 2010; 37 (5):3846–3852.

[15] Kantar E, Keskin M, Deviren B. Analysis of the effects of the global financial crisis on the Turkish economy, Using hierarchical methods. Physica A. 2012; 391 (7):2342–2352.

[16] Kantar E, Deviren B, Keskin M. Hierarchical structure of Turkey’s foreign trade. Physica A. 2011; 390 (20):3454–3476.

[17] Tabak BM, Serra TR, Cajueiro DO. Topological properties of stock market networks: the case of Brazil. Physica A. 2010; 389 (16):3240–3249.

[18] Huang FX, Zhao X, Hou TS. Hierarchy of the SSE 50 Index based on minimum spanning tree. Systems Engineering. 2009; 27 (1): 71–76.

[19] Yang R, Li XY, Zhang T. Analysis of linkage effects among industry sectors in China’s stock market before and after the financial crisis. Physica A. 2014; 411:12–20.

[20] Forbes KJ, Rigobon R. No contagion, only interdependence: measuring stock market co-movements. The Journal of Finance. 2002; 57:2223–2261.

[21] Engle RF. Dynamic conditional correlation-a simple class of multivariate GARCH models. Journal of BUSiness & Economic Statistics. 2002; 20:339–350.

[22] Caporale GM, Cipollini A, Spagnolo N. Testing for contagion: A conditional correlation approach. Journal of Empirical Finance. 2005; 12:476–489.

[23] Cappiello L, Engle RF, Sheppard K. Asymmetric dynamics in the correlations of global equity and bond returns. Journal of Financial Econometrics. 2006; 4:537–572.

[24] Chen K, Luo P, Sun BX, Wang HQ. Which stocks are profitable? A network method to investigate the effects of network structure on stock returns. Physica A. 2015; 436:224–235.

[25] Pollet J, Wilson M. Average correlation and stock market returns. Journal of Financial Economics. 2010; 96(3): 364–380.

[26] Zheng ZL, Liu YS, Wang WX. Average correlation and systematic risk: evidence from Chinese market. China Economic Quarterly. 2014; 13(3):1047–1063.

[27] Mantegna RN. Hierarchical structure in financial markets. The European Physical Journal B-Condensed Matter and Complex Systems. 1999; 11(1): 193–197.

[28] Hafner CM, Reznikova O. On the estimation of dynamic conditional correlation models. Computational Statistics & Data Analysis. 2012; 56:3533–3545.

[29] Freeman LC. Centrality in social networks: conceptual clarification. Social Networks. 1978; 1: 215–239.

[30] Sensoy A, Tabak BM. Dynamic spanning trees in stock market networks: The case of Asia-Pacific. Physica A. 2014; 414:387–402.

[31] Onnela JP, Chakraborti A, Kaski K, Kertész J. Dynamic asset trees and portfolio analysis. European Physical Journal B. 2002; 30:285–288.

[32] Flavin TJ, Panopoulou E, Unalmis D. On the stability of domestic financial market linkages in the presence of time-varying volatility. Emerging Markets Review. 2008; 9:280–301.

[33] Kuzubaş TU, Ömercikoğlu I, Saltoğlu B. Network centrality measures and systemic risk: An application to the Turkish financial crisis. Physica A. 2014; 405:203–215.

[34] Blundell R, Bond S. Initial conditions and moment restrictions in dynamic panel data models. Journal of Econometrics. 1998; 87:115–143.

